# Analysis of digital twin applications in nursing practice and education: a scoping review

**DOI:** 10.3352/jeehp.2026.23.13

**Published:** 2026-06-09

**Authors:** A Reum Lim, Hyun Kyoung Kim

**Affiliations:** 1 Department of Nursing, Kyeongbuk College of Health, Gimcheon, Korea; 2 Department of Nursing, Kongju National University, Gongju, Korea; Hallym University, Korea

**Keywords:** Artificial intelligence, Machine learning, Nursing education, Patient-centered care, Self-management

## Abstract

This scoping review examined research applying digital twins in nursing practice and education and summarized their application domains, methods, outcomes, and implications. A human digital twin is a virtual health replica modeled from real-world data. This study followed the 5-stage scoping review process proposed by Arksey and O’Malley. Two researchers independently conducted the literature search without restrictions on publication year. From April 1 to 15, 2026, the Cochrane Library, PubMed, Embase, CINAHL, ERIC, and RISS databases were searched, and 15 studies were ultimately included. Digital twin applications were identified in 3 major domains: clinical practice and patient-centered care, education and training, and decision-making and workflow management. Application methods and outcomes varied according to technological implementation and included (1) modeling and data-driven prediction, (2) development of immersive learning and practice-training environments, and (3) system integration and decision-support frameworks. In clinical settings, multimodal patient data can be analyzed using artificial intelligence and machine learning to generate a virtual persona resembling the patient, thereby facilitating real-time personalized nursing care and self-management. In educational settings, digital twins can provide realistic and safe learning environments that enhance training effectiveness. Digital twins show substantial potential to advance predictive and personalized nursing in both clinical practice and education. Their data-driven capabilities are expected to contribute to innovative applications in future nursing practice and educational environments.

## Graphical abstract

[Fig f3-jeehp-23-13]

## Introduction

### Background

Digital twin (DT) technology refers to an advanced system that replicates physical entities or processes from the real world in real time, enabling simulation, prediction, and intervention within a virtual environment [1]. By integrating artificial intelligence (AI), the Internet of Things (IoT), cloud computing, and big data analytics, DTs establish a continuous feedback loop between actual data and virtual models, making it possible to reproduce and forecast human health conditions precisely [2]. Originating in the aerospace and manufacturing industries, DTs have rapidly expanded into the medical and health sectors, where they are regarded as foundational infrastructure for advancing precision and predictive medicine [3]. A human DT is a virtual health replica that comprehensively models physiological, anatomical, and behavioral characteristics based on multimodal data [4]. DTs demonstrate strong potential for clinical application by incorporating real-time biosignals to monitor patient conditions, simulate treatment responses, and optimize the allocation of healthcare resources [5]. Thus, DTs are not merely automation tools but transformative frameworks that integrate data across patient, system, and policy levels to predict health outcomes and support a shift toward preventive health systems [6].

In healthcare education, DTs have recently gained attention as tools for personalized treatment planning, clinical decision support, and simulation-based training [7]. However, research in nursing remains at an early stage, and there is limited systematic understanding of how DTs are being applied in nursing education and clinical practice, as well as their effects and limitations. Emerging studies in nursing-related domains indicate that DTs have been used to improve workforce allocation and workload prediction, thereby enhancing unit operations and supporting leadership-driven decision-making in nursing management [8]. In clinical nursing, a DT-supported home care randomized controlled trial demonstrated improved hypertension management outcomes, including reduced antihypertensive medication needs and alleviation of high blood pressure [9]. A DT model predicting chemotherapy responses based on genomic information in patients with ovarian cancer contributed to personalized treatment support [10], while DT applications in patients with heart failure enabled home-based biosignal monitoring and prediction of deterioration, including fall and infection risks [11]. These findings collectively highlight the potential of DTs to enable prediction-driven nursing practice across administrative and clinical domains. Nevertheless, DT-related research remains predominantly centered in medical and engineering fields, with insufficient exploration of its conceptual significance, applicability, and implications from a nursing perspective.

### Objectives

This study aimed to map and summarize the utilization of DTs in nursing through a comprehensive scoping review. By examining DT use in nursing clinical practice, education, administration, and research, this study sought to identify current applications, propose future research directions, and evaluate practical implications. Specifically, this study reviewed research applying DTs in nursing practice and education and summarized the application domains, methods, outcomes, and implications of DT use.

## Methods

### Ethics statement

This was a literature-based study; therefore, neither institutional review board approval nor informed consent was required.

### Study design

This study was conducted according to the 5-stage scoping review framework proposed by Arksey and O’Malley [12]. The reporting of this study adhered to the PRISMA-ScR (Preferred Reporting Items for Systematic Reviews and Meta-Analyses extension for Scoping Reviews) guidelines [13].

### Eligibility criteria: Stage 1 (identifying the research questions)

To comprehensively examine how DTs are being used in nursing education and clinical practice, this study adopted the broad exploratory approach proposed by Arksey and O’Malley [12]. To formulate specific research questions, the Population–Concept–Context (PCC) framework recommended by the Joanna Briggs Institute (JBI) was applied. The Population included patients receiving care supported by DTs, nurses, hospitals, educational institutions, and community settings. The Concept encompassed DT technologies, models, interventions, curricula, and decision-making processes. The Context involved both clinical practice and educational environments. After iterative discussion and consensus among the researchers, the final research questions were established as follows: (1) In what domains are DTs applied within nursing? (2) How are DTs implemented in nursing, and what outcomes have been reported? and (3) What are the implications of DTs for nursing clinical practice and education?

The inclusion criteria were studies applying DT technology within the field of nursing or involving nurses, nursing students, or patients; peer-reviewed journal articles; full-text availability in English or Korean; and studies addressing DT implementation or discussing its nursing implications. The exclusion criteria were books, reports, conference proceedings, news articles, dissertations, and other forms of grey literature, as well as studies not directly related to DT technology or not situated within the nursing discipline.

### Information sources: Stage 2 (identifying relevant studies)

**Fig. 1.**
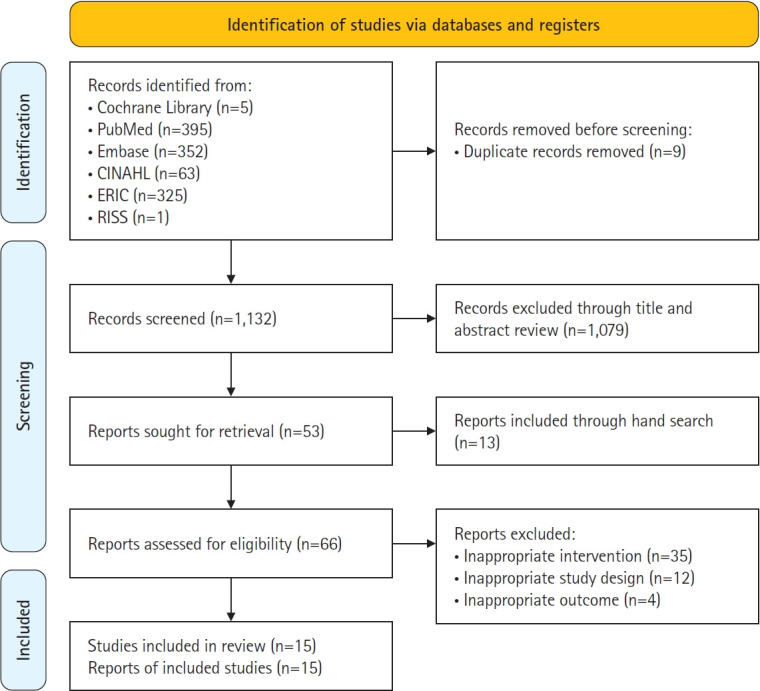
Flow chart of study selection.

The literature search was conducted independently by 2 researchers between April 1 and 15, 2026, without restrictions on publication year. The search strategy centered on the keywords “Digital Twin,” “Digital Twins,” “Digital Model,” “Virtual Twin,” “Virtual Patient,” and “Virtual Model.” These terms were combined with related concepts such as “nursing,” “healthcare,” “education,” “clinical,” “patient care,” and “simulation.” Both MeSH terms and free-text terms were applied and adapted to the indexing structure of each database (Supplement 1). Six databases were searched: 5 international databases—the Cochrane Library, PubMed, EMBASE, the Cumulative Index to Nursing and Allied Health Literature (CINAHL), and the Education Resources Information Center (ERIC)—and 1 Korean research database, the Research Information Sharing Service (RISS). In addition, manual searches were performed using Google Scholar. The database-specific search and selection processes are illustrated in the PRISMA-ScR flow diagram [13] (Fig. 1).

### Search strategy: Stage 3 (study selection)

A total of 1,141 records were identified through database searching: 5 from the Cochrane Library, 395 from PubMed, 352 from EMBASE, 63 from CINAHL, 325 from ERIC, and 1 from RISS. After 9 duplicates were removed, 1,132 titles and abstracts were screened. Of these, 1,079 records were excluded because they were unrelated to the research topic or were from fields outside nursing. Following the first screening, 53 studies were selected for full-text assessment. An additional 13 studies were identified through reference-list screening and manual searches using Google Scholar, resulting in 66 articles eligible for full-text review. Two reviewers independently assessed all 66 full-text articles and excluded 51 studies that involved interventional designs or did not meet the predefined eligibility criteria (Fig. 1).

### Data charting process: Stage 4 (charting the data)

For the 15 studies included in the final review, 2 researchers independently and systematically extracted key information. Data charting was conducted based on the procedures outlined in the JBI Reviewer’s Manual for scoping reviews [14]. A predefined, consensus-based data charting form was used to organize essential information from each study. Because this study was designed as a scoping review to map the range and characteristics of available evidence, formal methodological quality appraisal of individual sources was not conducted.

### Data items and synthesis methods: Stage 5 (analyzing the evidence)

The extracted items included author(s), year of publication, country, study design, study population or end user, DT application concept and technological components, implementation context, main findings, and nursing implications. All extracted data were compiled using Excel (Dataset 1). When discrepancies arose between the 2 researchers, they were resolved through mutual review and discussion until consensus was reached. Data analysis proceeded in alignment with the research questions, using a stepwise approach to compare and synthesize the content of each study. Through this process, DT application domains and themes, as well as the methods and effects of DT implementation, were identified. These analytic procedures enabled a systematic understanding of the current status and emerging trends in DT use within nursing.

## Results

### Study selection

All 15 studies included in the final review were published between 2021 and 2025 (Appendix 1). The largest proportion of studies was conducted in the United States (n=6) [A1,A2,A6,A9,A11,A14], followed by India (n=5) [A4,A5,A8,A10,A15].

### Results of syntheses

The study designs represented a diverse range of methodological and analytical approaches, including 4 machine learning studies [A4,A7,A10,A12], 3 simulation studies [A1,A3,A14], 2 randomized controlled trials (RCTs) [A5,A8], 2 proof-of-concept studies [A6,A9], 1 expert commentary [A2], 1 case study [A11], 1 proposed cohort study [A13], and 1 cohort study [A15]. The study end users included nursing administrators [A1-A3,A14], nurses caring for patients with diabetes [A8-A10,A15], nurses caring for patients with hypertension [A4,A5], oncology nurses [A6,A7,A12], gerontology nurses [A13], and nursing educators [A11]. All included studies addressed DT technology as a central concept [A1-A7]. Advanced technologies such as virtual reality (VR) [A11], discrete event simulation (DES) [A1,A3,A14], machine learning [A4,A10,A12], deep learning [A7], and knowledge graphs [A9] were integrated to enhance efficiency in nursing and healthcare environments and support data-driven decision-making processes (Table 1).

### Application domains of DTs

DTs were applied for a wide range of purposes within nursing practice and broader healthcare environments. The application domains were categorized into 3 areas: clinical practice and personalized patient care [A5-A10,A12,A13,A15], education and training [A2,A11], and decision-making and workflow management [A1,A3,A4,A14] (Table 1).

#### Clinical practice

DTs were utilized as tools to enhance efficiency in clinical settings, improve the quality of patient care, and enable personalized nursing tailored to patients’ individual characteristics and needs. They integrated lifestyle data from patients with diabetes [A8-A10] and hypertension [A4,A5] to develop personalized management models based on patient-specific characteristics. DT technology constructed virtual personas derived from real-world data from patients with cancer [A6,A7,A12], establishing a foundation for individualized information delivery tailored to both patients and caregivers. Collectively, these studies demonstrated the potential of DT technology to promote personalized nursing care and strengthen self-management capabilities by reflecting patient-specific profiles.

#### Education and training

DTs were employed as educational and training tools to enhance nurses’ and healthcare professionals’ clinical readiness and learning engagement. Carroll and Garcia-Dia [A2] highlighted the potential of DT technology to drive transformation across nursing leadership, clinical practice, and education. They emphasized the need for nursing leaders to establish data ethics and technology-use guidelines to prepare organizations for DT integration. Zackoff et al. [A11] developed an onboarding training program that combined DTs with VR, enabling clinical staff to become familiar with the layout and operational environment of new clinical spaces. In this domain, DTs served as key mechanisms for creating immersive simulation-based learning environments and strengthening digital competency–driven nursing education.

#### Decision-making and workflow management

DTs were also utilized as frameworks to support decision-making and optimize workflow processes within healthcare organizations [A4]. Kuruppu et al. [A3] developed a DES-based DT framework in a coronary care unit (CCU) setting to analyze bed turnover, staffing allocation, and resource utilization efficiency. Anyene et al. [A1] proposed a co-simulation-based DT framework to support healthcare planning and resource management decisions, virtually modeling collaboration between nurses and robotic systems to demonstrate improvements in workforce efficiency and patient-centered workflow design. Additionally, Pilati et al. [A14] applied DTs to the operation of coronavirus disease 2019 (COVID-19) vaccination centers, simulating staffing redistribution and vaccination processes to enhance operational efficiency in large-scale public health systems and propose predictive resource management models. Collectively, these studies demonstrated the effectiveness of DTs in supporting data-driven decision-making, visualizing operational processes, and improving workflow efficiency.

### Implementation methods and outcomes of DTs

The implementation methods of DTs varied depending on the purpose of each study, reflecting different levels of technological sophistication and application depth. Based on the mode of technological deployment, DT applications were classified into 3 categories: modeling and data-driven prediction [A3,A5-A10,A12,A13,A15], immersive learning and practice-training environments [A2,A11], and system integration and decision-support frameworks [A1,A4,A11,A14]. The major features and observed effects of each method are summarized below (Table 1).

#### Modeling and data-driven prediction

DTs were used to incorporate real-time clinical data to predict disease trajectories or resource flows and enhance the accuracy of healthcare decision-making. Sarani Rad et al. [A9] utilized a knowledge graph–based DT to analyze blood glucose data among patients with diabetes, enabling real-time prediction of glycemic fluctuations and provision of tailored lifestyle management strategies. Kuruppu et al. [A3] developed a DT framework grounded in DES to simulate and optimize bed turnover, staffing allocation, and resource utilization using real-time operational data. Similarly, Wang et al. [A6] constructed persona models that reflected the characteristics of patients with ovarian cancer and their families, establishing the foundation for developing personalized health information recommendation systems and demonstrating the feasibility of individualized information delivery. These modeling-based approaches collectively contributed to improved decision-making accuracy, enhanced personalization of patient care, and more efficient healthcare resource management.

#### Immersive learning and practice-training environments

Carroll and Garcia-Dia [A2] noted that DTs can be integrated into both prelicensure nursing education and continuing education for practicing nurses, enabling virtual simulation-based procedures without the need for real patients. Zackoff et al. [A11] developed an onboarding program that combined DTs with VR for clinical staff. Through this program, participants were able to familiarize themselves with the layout and procedures of new clinical environments before entering them, resulting in improved spatial awareness and readiness. These immersive learning approaches helped reduce uncertainty during transitions to new clinical settings and enhanced learner engagement and acceptance.

#### System integration and decision-support frameworks

Anyene et al. [A1] developed a co-simulation-based DT framework that replicated workflow interactions between intensive care unit nurses and collaborative robots, enabling analyses of staffing availability and changes in patient wait times. This approach demonstrated the potential to enhance workforce efficiency and support patient-centered decision-making. Pilati et al. [A14] constructed a DT model for COVID-19 vaccination centers to simulate staffing redistribution and operational scenarios, analyze variations in throughput, and propose strategies for improved decision support and operational efficiency. These system integration–based approaches contributed to greater predictability in healthcare administration, enhanced process efficiency, and the advancement of data-driven decision-making.

## Discussion

This scoping review included 15 studies published between 2021 and 2025, demonstrating that DT applications represent an emerging and rapidly developing concept within nursing. The studies encompassed a wide range of populations, including nursing leaders, healthcare professionals, and patients, and employed diverse research designs such as conceptual analyses, case studies, simulations, and proof-of-concept models. Across all studies, DTs served as the central technological concept, and their applications were observed primarily in clinical practice and nursing education. These findings suggest that DTs are gradually expanding across multiple domains of nursing, supporting a transition from nurses as passive technology adopters to active, data-driven decision-makers [15].

### Interpretation

**Fig. 2.**
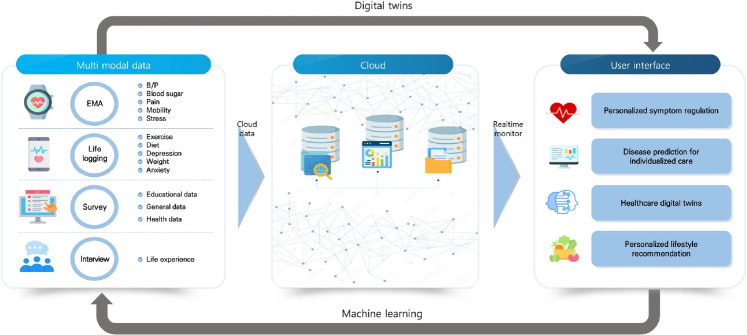
Architecture of digital twins.

Within the domain of patient care, DT-based persona models have been developed for patients with ovarian cancer and their families, offering the potential for personalized health information delivery and enhanced psychological support [10]. Among individuals with diabetes, knowledge graph–based DT models have been used to predict glucose levels and optimize insulin dosing, demonstrating the effectiveness of personalized diabetes management [16-18]. Although Woebot functions as a large language model-based conversational mental health assistant that directly interacts with real patients, DTs serve as multimodal, in silico patient models designed to simulate, predict, and analyze individuals’ physiological and behavioral conditions [19]. Although DT applications have not yet been validated in clinical trials, they have been proposed for long-term memory storage and AI-driven analysis of cardiovascular patient data [20], as well as the integration of multi-omics genomic datasets for patients with cancer [21], suggesting that similar applications could be adapted to nursing practice. DTs integrate multimodal patient data—including physiological measurements, environmental exposures, health behaviors, lifelogging information, survey data, and qualitative interviews—and apply machine learning analytics [22] to construct virtual personas that closely resemble actual patients. These virtual representations enable real-time personalized nursing care and support self-management (Fig. 2).

In the domain of clinical management, DT-based simulation of vaccination centers demonstrated improvements in resource allocation and staffing efficiency during the COVID-19 pandemic [23]. These findings indicate that DTs enable scenario-based decision-making for public health emergency responses and inform policy development related to nursing workforce management. DT simulation models in intensive care units [24] and broader hospital operations [8] further showed dual benefits: optimizing workflow and staffing structures while simultaneously enhancing nursing efficiency and patient safety. Such evidence supports a shift from experience-based staffing decisions to predictive management approaches for nurse administrators, demonstrating the potential of DTs to become a core tool for evidence-based nursing leadership and administration [25].

In the educational domain, the integration of DTs with virtual reality enabled successful immersive training in large-scale clinical space expansion scenarios, demonstrating improved efficiency in onboarding newly hired nurses and supporting organizational transitions during unit expansion [26]. This advancement suggests that nursing education can evolve beyond traditional knowledge transmission to experiential learning, offering realistic and safe simulated clinical environments that enhance learning outcomes and build learner confidence. As such, DTs are increasingly regarded as an innovative platform that integrates patient-centered care with data-driven nursing practice [27].

However, most existing studies remain at conceptual or technical stages, with limited empirical evidence grounded in nursing theory and insufficient exploration of ethical considerations and data governance frameworks [28]. Future research should examine the care-related implications of DTs through the lens of nursing theoretical frameworks and develop methodological approaches that integrate DT technology with core nursing concepts.

### Limitations

This review has several limitations. First, only English- and Korean-language publications were included, which may have excluded relevant studies in other languages. Second, grey literature, dissertations, conference proceedings, and technical reports were excluded, although DT applications are rapidly evolving in non-journal sources. Third, the included studies were heterogeneous in design, population, technological maturity, and outcome reporting, limiting direct comparison across studies. Fourth, because this was a scoping review, formal quality appraisal was not performed. Finally, several included studies were not nursing-specific but were interpreted according to their potential implications for nursing practice and education.

### Implications

DTs in nursing are being applied across multiple outcomes, including improving workflow efficiency and enhancing patient safety in clinical practice, increasing engagement and learning effectiveness in nursing education, enabling personalized interventions for patients with cancer or chronic diseases, and optimizing operations in public health and administrative settings. Strengthening nurses’ digital literacy and data interpretation competencies, as well as establishing interdisciplinary collaboration models, will be essential for advancing DT implementation in nursing practice.

## Conclusion

This scoping review suggests that DT technologies have emerging potential to support predictive and personalized nursing in clinical practice, education, and healthcare management. However, the current evidence remains limited, heterogeneous, and often indirect. Future studies should evaluate DT applications using nursing theoretical frameworks, rigorous empirical designs, and explicit ethical and data governance models.

## Figures and Tables

**Figure f3-jeehp-23-13:**
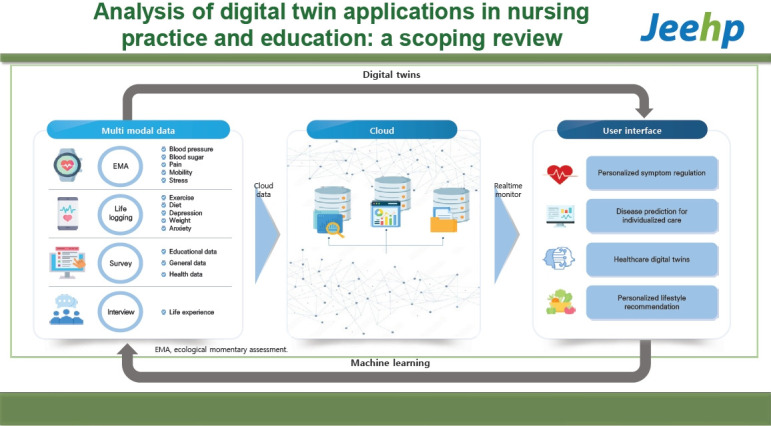


**Table 1.** Content and themes of selected studies (n=15)

No.	First author (year)	Country	Study design	End user	Domains	Methods	Findings	Implications
A1	Anyene (2025)	United States	Co-simulation framework study	Nursing administrator for ICU	DT co-simulation framework	Support for medical planning and resource management decision-making	Simulating the collaborative workflow between nurses and robots	Nursing workforce management and supporting patient-centered decision-making
A2	Carroll (2025)	United States	Expert commentary	Nursing leaders	Nursing practice	Clinical practice, process management, and education	Data management and ethical frameworks	Data technology capabilities, governance, and ethics
A3	Kuruppu (2025)	United Kingdom	Simulation study	Nursing administrator for CCU	Health DT framework for DES	Business process optimization and workflow monitoring	Real-time task tracking and resource utilization visualization	Support for designing efficient workforce and resource management
A4	Nair (2025)	India	DT decision modeling	Nurses monitoring BP	Inotropic infusion	Hypertension medication	Pharmaco-dynamic modeling	Patient safety and reduced workload
A5	Shamanna (2024)	India	RCT	Nurses monitoring BP	BP control	BP home monitoring	BP decrease	Effect of DTs on hypertension remission
A6	Wang (2024)	United States, Hong Kong	Proof-of-concept case study	Nurses caring for ovarian cancer	Customized treatment for patients with cancer	DT-based personalized information support	Health information recommendation system using personas	Patient-centered nursing and family involvement
A7	Bahrami (2023)	Switzerland	DT deep learning	Oncology nurses	Transdermal fentanyl therapy	Patient modeling for pain relief	Plasma fentanyl and ventilation rate	Effect of DTs on pain reduction
A8	Joshi (2023)	India	RCT	Nurses caring for diabetes	Fatty liver with diabetes	Personalized nutrition intervention	HbA1c decrease	Diabetes remission and decreased liver fat
A9	Rad (2023)	United States	Proof-of-concept study	Nurses caring for diabetes	Personalized diabetes management	Blood glucose prediction modeling	Personalized management using knowledge graphs	Patient self-management and continuous monitoring
A10	Thamotharan (2023)	India	DT deep learning	Nurses caring for diabetes	Diabetes management for older adults	Diabetes prediction	Patient modeling for diabetes	Personalized insulin infusion
A11	Zackoff (2023)	United States	Case study	Nursing educator	DTs with VR	Clinical space adaptation and team-based onboarding training	Enhanced immersion, spatial awareness, and clinical readiness	Adaptation to clinical environments and enhanced nursing education effectiveness
A12	Kim (2022)	South Korea	DT machine learning	Oncology nurses	Prostate cancer patient DT	Prediction of prostate cancer	Prediction of prostate cancer stage	Improved medical processes and health promotion
A13	Milne-Ives (2022)	United Kingdom	Proposal for cohort study	Nurses caring for older adults	DTs for multimorbidity	Retrospective cohort modeling	Identify lifetime risk factors for multimorbidity	Healthier aging and health management
A14	Pilati (2021)	Italy, United States	Simulation study	Nurses administering vaccines	DT-DES-based operational model for vaccination centers	Simulation for resource management in large-scale vaccination centers	Improved vaccination processing rates through workforce and resource allocation	Nursing management model during infectious disease outbreaks
A15	Shamanna (2021)	India	Retrospective cohort study	Nurses caring for diabetes	DT mobile application	Nutrition, sleep, and exercise intervention with coaching	Improvements in BMI, BP, and glucose indices	Improved diabetes management using artificial intelligence

ICU, intensive care unit; DTs, digital twins; CCU, coronary care unit; DES, discrete event simulation; BP, blood pressure; RCT, randomized controlled trial; HbA1c, hemoglobin A1c; VR, virtual reality; BMI, body mass index.

## Footnotes

**Data availability**
Data files are available from https://doi.org/10.7910/DVN/P6SEUA Dataset 1. List and characteristics of the 15 studies included in the analysis.

## Supplementary materials

Supplement 1. Search strategy.

Supplement 2. Audio recording of the abstract.

## Appendix 1. Articles included in the analysis

A1. Anyene G, Schultz C, Nepomuceno A, Kim I. Digital-twin co-simulation framework to support informed decision in healthcare planning and management. Simulation 2025;101:361-375. https://doi.org/10.1177/00375497241283047

A2. Carroll WM, Garcia-Dia MJ. Introducing digital twins into nursing practice. Nurs Manage 2025;56:13-16. https://doi.org/10.1097/nmg.0000000000000283

A3. Kuruppu Appuhamilage GDK, Hussain M, Zaman M, Ali Khan W. A health digital twin framework for discrete event simulation based optimised critical care workflows. NPJ Digit Med 2025;8:376. https://doi.org/10.1038/s41746-025-01738-4

A4. Nair VS, Niranga GH, Aryalakshmi CS, Sathyapalan DT, Madathil T, Pathinarupothi RK. Optimizing inotropic infusion with cluster specific ai decision models and digital twins. IEEE Access 2025;13:108316-108329. https://doi.org/10.1109/ACCESS.2025.3581969

A5. Shamanna P, Erukulapati RS, Shukla A, Shah L, Willis B, Thajudeen M, Kovil R, Baxi R, Wali M, Damodharan S, Joshi S. One-year outcomes of a digital twin intervention for type 2 diabetes: a retrospective real-world study. Sci Rep 2024;14:25478. https://doi.org/10.1038/s41598-024-76584-7

A6. Wang Y, Thaker K, Hui V, Brusilovsky P, He D, Donovan H, Lee YJ. Utilizing digital twin to create personas representing ovarian cancer patients and their families. Stud Health Technol Inform 2024;315:754-756. https://doi.org/10.3233/SHTI240314

A7. Bahrami F, Rossi RM, De Nys K, Defraeye T. An individualized digital twin of a patient for transdermal fentanyl therapy for chronic pain management. Drug Deliv Transl Res 2023;13:2272-2285. https://doi.org/10.1007/s13346-023-01305-y

A8. Joshi S, Shamanna P, Dharmalingam M, Vadavi A, Keshavamurthy A, Shah L, Mechanick JI. Digital twin-enabled personalized nutrition improves metabolic dysfunction-associated fatty liver disease in type 2 diabetes: results of a 1-year randomized controlled study. Endocr Pract 2023;29:960-970. https://doi.org/10.1016/j.eprac.2023.08.016

A9. Sarani Rad F, Hendawi R, Yang X, Li J. Personalized diabetes management with digital twins: a patient-centric knowledge graph approach. J Pers Med 2024;14:359. https://doi.org/10.3390/jpm14040359

A10. Thamotharan P, Srinivasan S, Kesavadev J, Krishnan G, Mohan V, Seshadhri S, Bekiroglu K, Toffanin C. Human digital twin for personalized elderly type 2 diabetes management. J Clin Med 2023;12 https://doi.org/10.3390/jcm12062094

A11. Zackoff MW, Rios M, Davis D, Boyd S, Roque I, Anderson I, NeCamp M, Gardner A, Geis G, Moore RA. Immersive virtual reality onboarding using a digital twin for a new clinical space expansion: a novel approach to large-scale training for health care providers. J Pediatr 2023;252:7-10. https://doi.org/10.1016/j.jpeds.2022.07.031

A12. Kim JK, Lee SJ, Hong SH, Choi IY. Machine-learning-based digital twin system for predicting the progression of prostate cancer. Appl Sci 2022;12:8156. https://doi.org/10.3390/app12168156

A13. Milne-Ives M, Fraser LK, Khan A, Walker D, van Velthoven MH, May J, Wolfe I, Harding T, Meinert E. Life course digital twins-intelligent monitoring for early and continuous intervention and prevention (LifeTIME): proposal for a retrospective cohort study. JMIR Res Protoc 2022;11:e35738. https://doi.org/10.2196/35738

A14. Pilati F, Tronconi R, Nollo G, Heragu SS, Zerzer F. Digital twin of COVID-19 mass vaccination centers. Sustainability 2021;13:7396. https://doi.org/10.3390/su13137396

A15. Shamanna P, Joshi S, Shah L, Dharmalingam M, Saboo B, Mohammed J, Mohamed M, Poon T, Kleinman N, Thajudeen M, Keshavamurthy A. Type 2 diabetes reversal with digital twin technology-enabled precision nutrition and staging of reversal: a retrospective cohort study. Clin Diabetes Endocrinol 2021;7:21. https://doi.org/10.1186/s40842-021-00134-7
