# Supplementary material for: Analysis of digital twin applications in nursing practice and education: a scoping review
Source: J Educ Eval Health Prof. 2026 Jun 9;23:13. doi: 10.3352/jeehp.2026.23.13 (PMC13433139; doi:10.3352/jeehp.2026.23.13)
Supplement: Supplementary file 1 [file jeehp-23-13-suppl1.docx]

**Supplement 1.** Search strategy

| Database | No. of article/included | Publication year | Advanced search |
| --- | --- | --- | --- |
| PubMed | 395/2 | 1995–2025 | (("Digital Twin"[tiab] OR "digital twins"[tiab] OR "Digital Model"[tiab] OR "Virtual Patient"[tiab] OR "Virtual Twin"[tiab] OR "Virtual Model"[tiab]) AND ("Healthcare"[Mesh] OR "Health"[tiab] OR "Medicine"[tiab] OR "Nursing"[tiab] OR "Clinical"[tiab] OR "Patient Care"[tiab])) |
| Embase | 352/15 | 1995–2025 | ('digital twin'/exp OR 'digital twin':ti,ab OR 'digital twins':ti,ab OR 'virtual patient':ti,ab OR 'virtual model':ti,ab OR 'virtual twin':ti,ab OR 'digital model':ti,ab) AND ('health care'/exp OR healthcare:ti,ab OR medicine:ti,ab OR nursing:ti,ab OR clinical:ti,ab OR 'patient care':ti,ab) |
| Cochrane Library | 5/0 | 2020–2022 | ("digital twin":ti,ab,kw OR "digital twins":ti,ab,kw OR "virtual patient":ti,ab,kw OR "virtual twin":ti,ab,kw OR "virtual model":ti,ab,kw OR "digital model":ti,ab,kw) AND ("healthcare":ti,ab,kw OR "health care":ti,ab,kw OR medicine:ti,ab,kw OR nursing:ti,ab,kw OR "clinical":ti,ab,kw OR "patient care":ti,ab,kw) |
| CINAHL complete | 63/6 | 2015–2025 | ((MH "digital twins") OR "digital twin" OR "digital twins" OR "virtual patient" OR "virtual twin" OR "virtual model" OR "digital model" ) AND ( (MH "Health Care+") OR healthcare OR "health care" OR medicine OR nursing OR clinical OR "patient care" ) |
| ERIC | 325/0 | 2006–2025 | ((MH "digital twins") OR "digital twin" OR "digital twins" OR "virtual patient" OR "virtual twin" OR "virtual model" OR "digital model" ) AND ( (MH "Health Care+") OR healthcare OR "health care" OR medicine OR nursing OR clinical OR "patient care" ) |
| RISS | 1/1 | 2024 | ‘디지털 트윈‘ ’간호‘ |
